# Differential effects of ageing on the neural processing of speech and singing production

**DOI:** 10.3389/fnagi.2023.1236971

**Published:** 2023-09-04

**Authors:** Nella Moisseinen, Teppo Särkämö, Jaakko Kauramäki, Boris Kleber, Aleksi J. Sihvonen, Noelia Martínez-Molina

**Affiliations:** ^1^Cognitive Brain Research Unit, Department of Psychology and Logopedics, Faculty of Medicine, Centre of Excellence in Music, Mind, Body and the Brain, University of Helsinki, Helsinki, Finland; ^2^Centre for Music in the Brain, Department of Clinical Medicine, Faculty of Health, Aarhus University, Aarhus, Denmark; ^3^School of Health and Rehabilitation Sciences, Centre for Clinical Research, University of Queensland, Brisbane, QLD, Australia; ^4^Department of Neurology, Helsinki University Hospital, Helsinki, Finland; ^5^Department of Information and Communication Technologies, Centre for Brain and Cognition, University Pompeu Fabra, Barcelona, Spain

**Keywords:** ageing, language, music, vocal production, fMRI, hemispheric laterality

## Abstract

**Background:**

Understanding healthy brain ageing has become vital as populations are ageing rapidly and age-related brain diseases are becoming more common. In normal brain ageing, speech processing undergoes functional reorganisation involving reductions of hemispheric asymmetry and overactivation in the prefrontal regions. However, little is known about how these changes generalise to other vocal production, such as singing, and how they are affected by associated cognitive demands.

**Methods:**

The present cross-sectional fMRI study systematically maps the neural correlates of vocal production across adulthood (*N*=100, age 21–88 years) using a balanced 2x3 design where tasks varied in modality (speech: proverbs / singing: song phrases) and cognitive demand (repetition / completion from memory / improvisation).

**Results:**

In speech production, ageing was associated with decreased left pre- and postcentral activation across tasks and increased bilateral angular and right inferior temporal and fusiform activation in the improvisation task. In singing production, ageing was associated with increased activation in medial and bilateral prefrontal and parietal regions in the completion task, whereas other tasks showed no ageing effects. Direct comparisons between the modalities showed larger age-related activation changes in speech than singing across tasks, including a larger left-to-right shift in lateral prefrontal regions in the improvisation task.

**Conclusion:**

The present results suggest that the brains’ singing network undergoes differential functional reorganisation in normal ageing compared to the speech network, particularly during a task with high executive demand. These findings are relevant for understanding the effects of ageing on vocal production as well as how singing can support communication in healthy ageing and neurological rehabilitation.

## Introduction

The brain ages across adulthood (Walhovd et al., 2011; Vinke et al., 2018). Following early models on age-related functional reorganisation, current research links cognitive ageing trajectories to a complex interplay of normal structural reorganisation and accelerating or mediating factors, such as ageing-related hearing loss (Eckert et al., 2012; Bennett and Madden, 2014; see also Andrews-Hanna et al., 2007), genetic determinants, and lifestyle (Kaufmann et al., 2019; see also Reuter-Lorenz and Park, 2014). Adapting to these parallel processes, the ageing brain may express multiple functional changes, including activation increases and neural dedifferentiation (for reviews, see Grady, 2012; Cabeza et al., 2018).

A core example of supportive reorganisation is the increasing bilateral (Cabeza, 2002) activation of the prefrontal regions during cognitively engaging tasks (Davis et al., 2008; see also Maillet and Rajah, 2013; Reuter-Lorenz and Park, 2014) such as rapid word production (Wierenga et al., 2008; Hoyau et al., 2017). However, it remains unclear how neurocognitive ageing impacts speech at large (for reviews, see Shafto and Tyler, 2014; Peelle, 2019). Some evidence suggests that ageing may selectively impact speech production rather than comprehension (Amunts et al., 2020; Taler et al., 2020; for review, see Shafto and Tyler, 2014), while others propose that these effects may relate to associated cognitive tasks (Davis et al., 2014). Moreover, while cognitive task demands can diversify the neural activation patterns of speech already at young age, for instance, during improvisation and recall (Liu et al., 2012; for review, see Beaty et al., 2016), the impact of neurocognitive ageing across different task demands in speech remains largely unknown. In determining these effects, it would be informative to compare speech to another auditory-motor vocal domain, focusing on functions that typically show early signs of decline, such as memory and fluid ability (see Samu et al., 2017).

Singing provides a viable comparison in this regard, as it elicits partially overlapping (for review, see Peretz et al., 2015) yet also more extensive bilateral activation in frontal and temporal regions in the young adult brain (Callan et al., 2006; Özdemir et al., 2006). This initially higher bilateralisation in singing raises the question of whether such an age-related increased frontal bilateralisation, as that observed in speech, would also appear with ageing in singing. Further, previous work has shown transfer effects from singing to language-related functions, such as verbal memory and fluency, in both healthy ageing (for review, see Román-Caballero et al., 2018) and neurological disorders, including aphasia, in which patients may partially retain their ability to sing (Martínez-Molina et al., 2022; Marchina et al., 2023). Determining whether the age-related functional changes impact both speech and singing or are selective to one modality can enhance our understanding of how ageing affects the broader domain of vocal production. In addition, it would inform us about how singing can have positive effects on speech and cognitive function in healthy and pathological ageing.

Using a large sample (*N* = 100; age range 21–88 years), this fMRI study investigates speech and singing processing across adulthood with three task pairs varying in their cognitive demands: (1) simple *Repetition* of familiar proverbs and song phrases, (2) cued *Completion* of familiar proverbs and song phrases from memory, and (3) cued *Improvisation* of new proverbs and song phrases. To avoid performance bias from memorizing study materials for the six-task design, we used commonly known, age-balanced naturalistic items in Repetition and Completion tasks. For uniformity, new items for Improvisation task were matched with familiar items in Completion. We hypothesized that (i) singing shows generally less extensive ageing effects than speech with regard to lateralisation, (ii) the Repetition and Completion tasks, utilizing highly familiar memory items and thus low cognitive demands, will produce similar ageing effects reflecting motor production rather than cognitive effort, and (iii) the Improvisation task, placing highest demands on prefrontal executive and working memory processing, will produce increasingly bilateral overactivation in speech relative to singing with advancing age.

## Materials and methods

### Participants

One hundred volunteers (55 female), aged 21–88 years (mean 49.2, SD 17.5), with normal or corrected to normal sight were recruited for this fMRI study. All participants were right-handed native Finnish-speakers (4 bilingual) with no diagnosis of a hearing impairment, language or neurological disorder, cognitive decline, or dementia. Participants had no professional background in music. Sixty-four reported engaging at least bi-weekly in amateur-level music activities, fifty-nine of whom singing (solo, choir or other form of group singing). All participants were pre-screened for contraindications for MRI upon recruitment and again immediately before scanning. Prior to participation, all participants provided written informed consent to participate and to the use of their data for the scientific purposes of this study. The study was approved by the European Research Council Executive Agency (ERCEA) and the University of Helsinki Ethical Review Board in the Humanities and Social and Behavioural Sciences.

### Materials

#### Preselection and matching of naturalistic and new materials

As learning and memory functions decline with normal ageing, the present study used age-balanced naturalistic stimuli to prevent bias from memorisation of study materials. Candidate items for the Repetition and Completion tasks (40 proverbs +40 songs) were pre-selected for a pilot questionnaire from collections of commonly known proverbs and songs. Candidate selection prioritized items of Finnish origin to avoid interference from other language versions and was restricted in length to minimise variability in the final stimulus set. Syllable count was used as the main measure of length because compound words and suffixes are typical for Finnish language: using word count might lead to remarkable differences in phrase length. The song material comprised old children’s songs and nursery rhymes, folk songs, evergreens, Finnish pop/rock classics, and seasonal songs. Song phrases were selected either from the beginning of the first verse or chorus, avoiding internally repetitive, non-unique phrases and non-lexical utterances such as “ooh.”

The familiarity of all candidate items was tested with an online pilot questionnaire using a 4-point Likert scale (1 = completely unfamiliar, 4 = extremely familiar). Specifically, respondents were instructed to report their personal feeling of familiarity with each item, emphasising that the questionnaire was not a test but aimed at providing information about which proverbs and song phrases were most and least familiar at the population level. There were also five fabricated control items (non-existing proverbs/song phrases) in each category to validate the reliability of the ratings. To control for potential age differences in familiarity, the respondents of the pilot questionnaire (*N* = 665) were divided into three age groups (20–39 years, *N* = 246; 40–59 years, *N* = 294; 60 and above, *N* = 125) and 20 proverbs +20 song phrases per task were selected from the items rated most familiar across all age groups.

Items constructed for the Improvisation tasks were carefully matched to the selected Completion task items in phrase length, sentence structure, semantic theme, as well as for musical style in the Song Improvisation task. Selected items were recorded in female and male voice, matching all items in duration, tone, and expression. Recordings were carried out by a professional female and male musician who also composed the melodies for Song Improvisation items.

#### Speech and song stimuli

Stimuli consisted of familiar proverbs and song phrases for Repetition, beginnings of familiar proverbs and song phrases for Completion, and beginnings of new proverbs and song phrases for Improvisation; a total of 20 proverb +20 song phrase items in each task. After familiarity-based final selection, phrase lengths across both modalities remained similar, with song phrase items compared to proverb items being longer by 0.5 words / 1 syllable across all tasks (see Table 1). Melodic production in singing resulted in longer durations compared to speech. In addition, Completion and Improvisation items, containing only the first half of a phrase, were shorter than Repetition items which contained the full phrase.

**Table 1:** Stimulus items in speech (proverb) and singing (song phrase) tasks.

Task and item	Words	Syllables	Duration (ms)
**Repetition**			
Proverb (stimulus)	3.70 (0.66)	8.80 (1.61)	2,289 (280)
Song (stimulus)	4.60 (0.88)	10.00 (1.65)	5,055 (1371)
**Completion**			
Proverb (stimulus)	2.85 (0.59)	6.30 (1.13)	1,776 (352)
Proverb (target)	2.85 (0.49)	6.35 (1.14)	–
Song (stimulus)	3.20 (0.77)	7.40 (1.47)	3,168 (812)
Song (target)	3.25 (0.79)	7.15 (1.14)	–
**Improvisation**			
Proverb (stimulus)	2.85 (0.59)	6.50 (1.43)	1,849 (247)
Song (stimulus)	3.10 (0.72)	7.40 (1.35)	3,082 (499)

Lengths and durations of naturalistic items selected for repetition and completion tasks, and of new phrases for improvisation task, formulated to match with the completion task stimuli. All values reported as mean (SD).

### Experimental design and statistical analysis

The neural substrates of vocal production in speech and singing were investigated with a 2 × 3 cued task design involving two modalities [speech (Proverb) and singing (Song)] and three conditions (Repetition, Completion, and Improvisation). Each task consisted of 20 cue-response pairs. In Repetition, participants heard a highly familiar cue (full one-part proverb or song phrase) and then repeated it. In Completion, the cue was the beginning of a highly familiar proverb or song phrase, and the task was to produce the ending. For example, the correct response to cue ‘*An apple a day…*’ would be:’*…keeps the doctor away.*’ In Improvisation, the cue was the beginning of a new proverb or song phrase to which the participants were asked to improvise an ending by spontaneous completion of the verbal expression: by speech in Proverb and by singing (with melody and lyrics) in Song. Thus, the three conditions systematically explored different cognitive demands from simple motor production and short-term verbal working memory (Repetition) to additional components involving retrieval from long-term memory (Completion) and cognitive flexibility / fluency of spontaneous production (Improvisation). All tasks were in Finnish.

#### Procedure

Recruited participants reported their familiarity with memory task stimuli (Repetition items and cue parts of the Completion items) as a part of a comprehensive questionnaire battery assessing demographic information, health, and wellbeing as well as personal history with various leisure activities, including music (listening, singing, playing, and dancing). Although memory effects as such were outside the scope of this study, familiarity ratings were nevertheless expected to cause some memory activation. Participants were thus allowed to review the questionnaire items before scanning to balance out inter-individual differences from variable rating-scanning delays.

Prior to scanning, participants received instructions on a computer screen along with three practice trials mimicking the course of a task in the fMRI experiment. The researcher ensured that the participant understood the tasks and was able to carry them out. During scanning, the researcher informed the participant about which of the six tasks was about to begin and ensured that the instructions for the task were clear. For each task, participants were instructed to try their best and to always speak in the Proverb tasks and sing in the Song tasks.

Task order was pseudo-randomised in such a way that neither modality was presented twice in a row, no task type (Repetition / Completion / Improvisation) was presented twice in a row, and the first task rotated. Task order was counter-balanced between young (aged 20–39 years), middle-aged (40–59) and old (60–90) participants and between participants with regular (at least once per week for at least 1 year) vs. no singing activities. Stimuli were presented in random order within each task.

#### MR data acquisition

Data were acquired at the Advanced Magnetic Imaging (AMI) Centre of Aalto University, Espoo, Finland, with a MAGNETOM Skyra 3.0 T scanner (Siemens) using a 32-channel RF receiving head coil. Participant’s head was supported in place with soft foam paddings inside the coil to reduce head movement and protect hearing. Participants were asked to report immediately should they experience any discomfort or difficulty in perceiving instructions or stimuli. A whole-brain T1-weighted anatomical volume for anatomic normalisation was obtained using a 3D magnetisation-prepared rapid gradient-echo (MPRAGE) sequence: echo time (TE) = 3.3 ms, repetition time (TR) = 2530.0 ms, flip angle (FA) = 7 degrees, voxel size = 1 × 1 × 1 mm^3^, FOV = 256 mm and slices per slab = 176. T2*-weighted functional imaging was carried out using gradient echo-planar imaging (EPI) on the whole brain: TE = 30.0 ms, FA = 90 degrees, repetition time (TR) = 6,800–10,800 ms, delay in TR = 6,110–10,110 ms, voxel size = 3 × 3 × 3 mm^3^, FOV = 240 mm, and slices per volume = 44.

**Figure 1:**
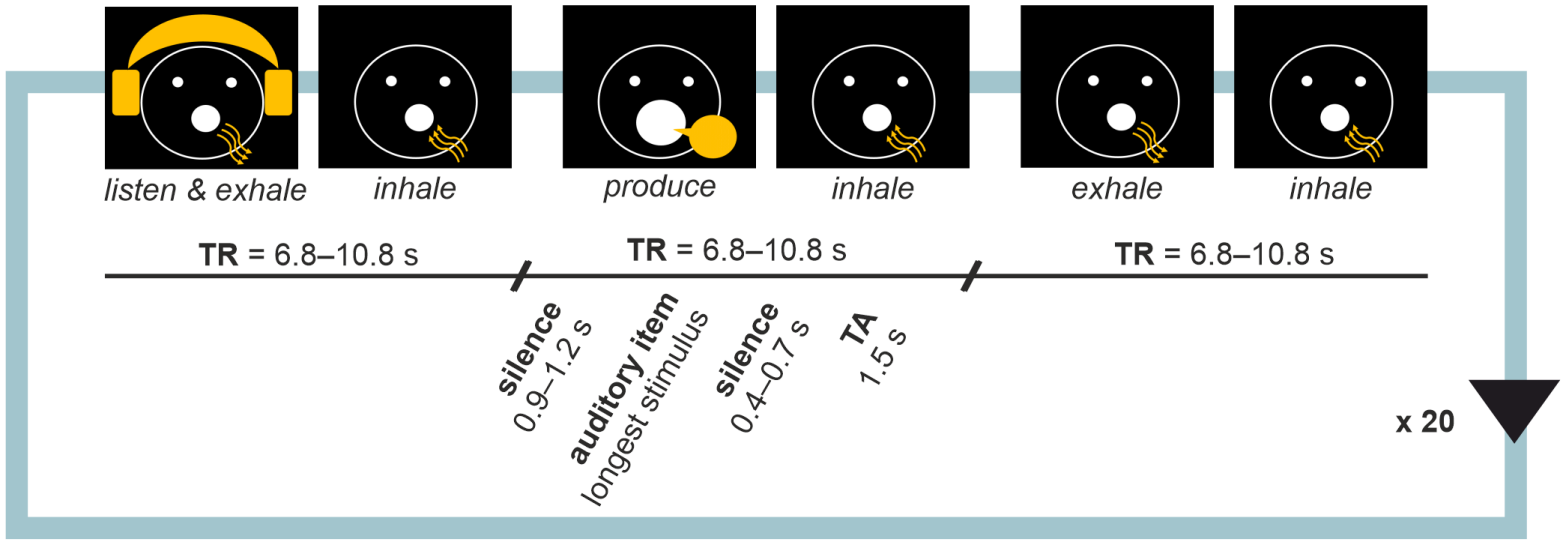
Sparse sampling block design. Each task involved 20 repetitions of a listen-produce-baseline trial triads. Activities for the participant were instructed with visual cues where arrows indicated the direction of airflow in breathing. Each activity was followed by inhalation during which volume acquisition occurred. Participant’s voice was recorded during production trials for quality control.

Before each measurement, sound volume was adjusted to a clearly audible but comfortable level, and microphone position was adjusted as per sound quality tests as needed. Visual instructions for active conditions were presented as graphical icons on a back projection mirror screen during tasks (see Figure 1). Auditory stimuli were presented using MR-compatible high-quality binaural insert earphones (KAR ADU2a). Vocal responses were recorded with a high-dynamic-range noise-cancelling optic microphone (Optoacoustics FOMRI-III) attached to the head coil. The tasks were created and instructions and stimuli were presented with Presentation 20.3 (Neurobehavioural systems, Inc., Berkeley, CA, United States) using MR pulses as triggers.

Each task involved 20 visually cued active condition triads: (i) listen and exhale, (ii) respond, and (iii) exhale (baseline), with inhale periods in between. A sparse-sampling technique (Hall et al., 1999; see also Belin et al., 1999; Yang et al., 2000) was adopted with silent intervals between consecutive whole-brain volume scans (see Figure 1). Each trial began with a long silent period (900–1,200 ms), followed by the active condition (length dependent on task), a short silent period (400–700 ms) and volume acquisition time window (1,500 ms). Since stimulus durations varied as per experiment design, times of repetition (TRs) were optimised task by task, resulting in TR = 6,800 ms in Proverb Repetition, 7,000 ms in Proverb Completion, 7,000 ms in Proverb Improvisation, 10,800 in Song Repetition, 8,800 ms in Song Completion and 8,000 ms in Song Improvisation. Systematic variation (jittering) of the timings of the active trials aimed for acquiring the peak of the hemodynamic response, estimated to occur with a 4–7 delay (Belin et al., 1999; Yang et al., 2000). Each task comprised 20 blocks of trial triads plus a start trial involving the initial inhalation, 61 trials in total.

#### MR preprocessing and first-level model

Functional MR volumes were pre-processed with Statistical Parametric Mapping (SPM) 12 software package (The Wellcome Centre for Human Neuroimaging, UCL, London) running under Matlab 2019a (The MathWorks Inc., Natick, MA, United States). EPIs were realigned to the first scan by estimating the parameters of an optimal rigid body transformation. The mean and individual EPIs were also manually reoriented to the anterior commissure to improve co-registration with the T1-weighted (T1w) image. After co-registration of the T1w with the mean EPI as reference, segmentation was performed using unified segmentation (Ashburner and Friston, 2005) involving medium regularisation and light clean-up with SPM12 IXI data set tissue probability maps. All EPIs were normalized to the MNI template by applying the spatial normalisation parameters from the T1w image and resampling to 2-mm isotropic voxels using trilinear interpolation. As vocal production tasks were expected to cause head movement above that of an average tb-fMRI experiment, the Artifact Detection Toolbox (Mozes and Whitfield-Gabrieli, 2011; available at http://www.nitrc.org/projects/artifact_detect/) was run on the unsmoothed EPIs to identify potential outlier scans using a 3-mm framewise displacement threshold with global BOLD signal change threshold of 4 SD. Participants displaying a within-task outlier scan rate of 20% or above were rejected. Finally, EPIs were spatially smoothed using spatial convolution with an 8-mm FWHM Gaussian kernel to minimize effects of inter-individual anatomical differences.

Main effects were estimated with the general linear model (GLM) framework. Each condition was modelled with a boxcar function convolved with the canonical hemodynamic response function (HRF), as this has been shown to yield the best model fitting in sparse-sampling acquisitions (Perrachione and Ghosh, 2013). Based on previous literature (Belin et al., 1999; Kleber et al., 2010), we opted for a ‘slow’ event-response analysis and set trial duration to 0 s. Microtime resolution was adjusted to 125 ms per bin corresponding to the standard value used in continuous fMRI, with middle slice as the reference. A high-pass filter with a 128 s cut-off was applied to remove slow signal drifts. Temporal autocorrelations in the BOLD signal time series were accounted for with an AR (1) model to approximate the observed covariance of the functional data in the context of restricted maximum likelihood estimation. We included the TPM for grey matter tissue thresholded at 15% probability as explicit mask. No global scaling was applied. In addition to experimental condition effects, the design matrix included nuisance regressors to control for the effects of head motion (6 realignment parameters) and potential outlier scans (one regressor per outlier scan; Jenkinson et al., 2002). The model was first estimated within each subject, and contrast-of-interest volumes were generated comparing the appropriate conditional coefficients to assess the main effects of production versus exhalation, which served as the baseline condition.

#### Stimulus familiarity and performance assessment

Performance in the fMRI tasks was assessed in terms of response timing (all tasks) as well as stimulus familiarity ratings and response match with target (Repetition and Completion tasks) based on measurement documentation (filled-in log sheets) and recorded vocal responses. Given that the sample consisted of nonmusicians, with approximately half reporting no singing experience, and considering that singing skill and performance were not the focus of this study, we placed a greater emphasis on verbal accuracy over melodic accuracy when assessing sung responses in the Repetition and Completion tasks. In these tasks, deviations from target responses were marked as erroneous, while allowing for non-lexical utterances such as ‘um’ or ‘haha’ as fillers and replacing words with synonyms. To harmonize the rising of the BOLD responses across trials, timing was marked as erroneous should the response begin too early or end too late with more than 25% of the response falling outside the production time window. A technical failure in the laboratory resulted in poor audio recording quality for 26 participants, due to which errors documented during measurements could not be verified from recorded vocal responses. For these participants, performance was only assessed with measurement documentation.

#### MR group-level model and laterality index

Group effects were studied with (i) main effects across the age range, (ii) ageing effects, and (iii) ageing effects on laterality index (LI). First, as the paradigm allowed controlling for inhalation-related activation implicitly, production versus baseline (exhale) contrasts (Repetition > Baseline, Completion > Baseline, Improvisation > Baseline) from each participant were used directly to calculate the main effects of each task with one-sample t-tests. Direct modality comparisons (Proverb > Song, Song > Proverb) within each task type (Repetition, Completion, Improvisation) were calculated with paired t-tests. Ageing effects for speech, singing, and their within-subject difference were studied with multiple regression in both directions (i.e., increasing or decreasing with advancing age) for the whole brain. All models controlled for variation in education years, age-relative experience with musical hobbies (% of age active in musical hobbies in years) and total intracranial volume (TIV). These were supplemented with age for studying the main effects of each task (*t*-tests).

In addition, age-related laterality shifts [as predicted by the Hemispheric Asymmetry Reduction in Older Adults (HAROLD) model; (Cabeza, 2002)] were studied independently with 25-percent trimmed mean scores from bootstrapped laterality indices, a histogram-based fMRI adaptation of the classical approach


$$LI=\frac{\sum {\mathrm{activation}}_{\mathrm{LEFT}}-\sum {\mathrm{activation}}_{\mathrm{RIGHT}}}{\sum {\mathrm{activation}}_{\mathrm{LEFT}}+\sum {\mathrm{activation}}_{\mathrm{RIGHT}}}$$

(LI toolbox for SPM; Wilke and Schmithorst, 2006; Wilke and Lidzba, 2007), on the whole brain (grey matter) with stepwise multiple linear regression in SPSS (IBM SPSS Statistics 28). Unfortunately, as the calculation of LI aborted if the minimum bootstrapped (within-subject) sample size was not reached, group-level sample sizes were partially reduced in LI analyses. In addition to whole-brain analyses, laterality changes in pre-defined regions of interest (ROIs), derived from the probabilistic connectivity-based Brainnetome atlas (available at https://atlas.brainnetome.org), were analysed for task pairs where age-associated functional reallocation showed a significant laterality change at larger scale. To test for the previously established laterality shift in the (pre-) frontal regions (Cabeza, 2002; see also Wierenga et al., 2008; Hoyau et al., 2017) and whether such an effect would also appear in temporal and parietal auditory-motor vocal production regions (Callan et al., 2006; Özdemir et al., 2006), this fine-grained approach involved MFG (Brainnetome subregions 15–28), IFG (29–40), orbitofrontal (OFG; 41–52) and precentral gyri (PrCG; 53–64) frontally; STG (69–80), middle temporal gyri (MTG; 81–88), inferior temporal gyri (ITG; 89–102), posterior superior temporal sulci (pSTS; 121–124) temporally; and inferior parietal lobules (IPL; 135–146) and postcentral gyri (PoCG; 155–162) parietally. In the stepwise regression, education years, musical hobbies, and TIV were introduced as independent variables in addition to age. Alpha levels (0.05) were corrected for multiple comparisons at each level of testing (Bonferroni).

## Results

### Demographic characteristics and performance of participants

**Figure 2:**
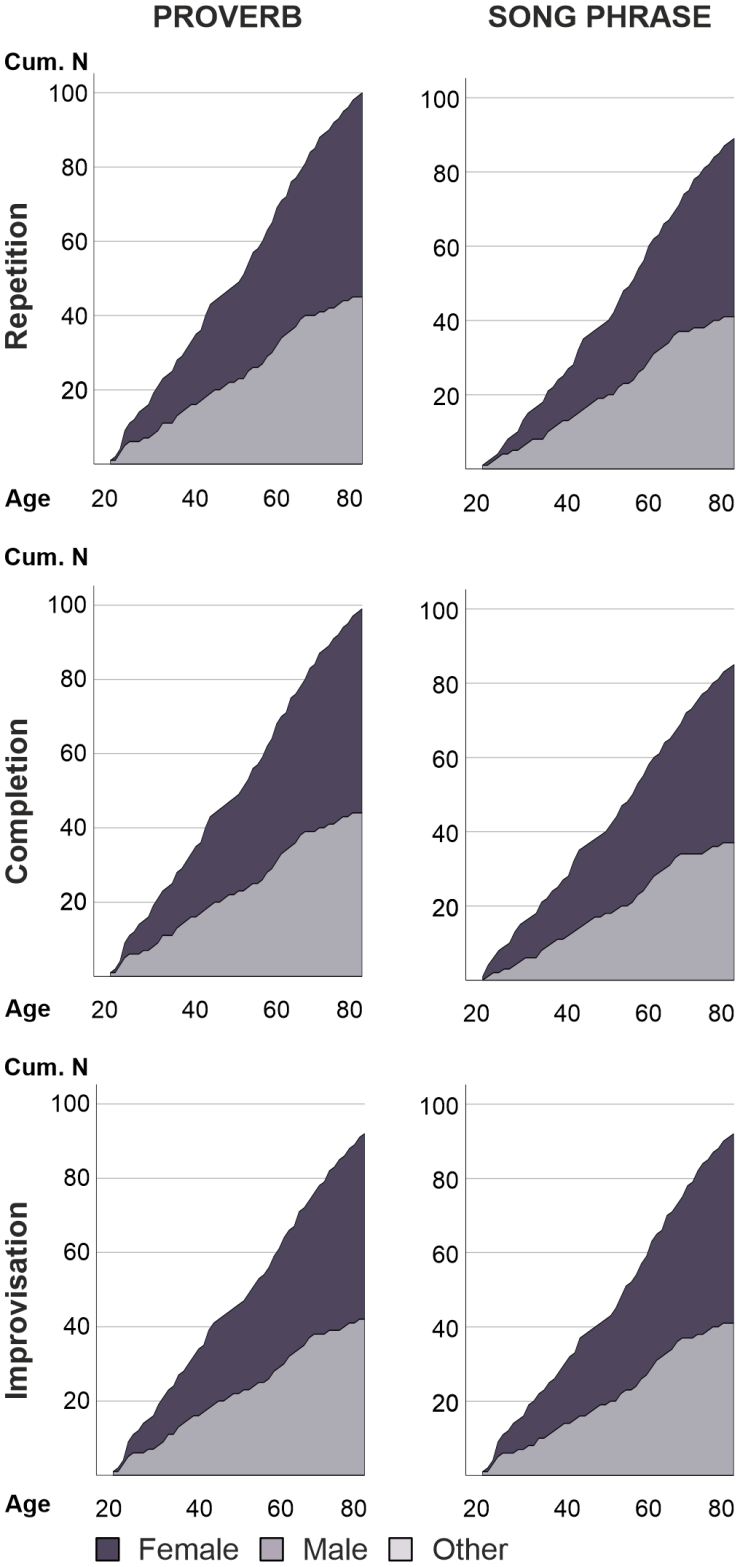
Cumulative age and gender distributions. Age distributions of the accepted participants by task. Dark, middle, and light grey tones within the distributions represent female, male, and other genders, respectively.

After participation, all data was inspected for deviations in quality or vocal production performance. As the purpose of the present study did not involve direct comparisons between the task types (Repetition, Completion, Improvisation), this was done task-by-task to maximise the sample size for each condition. Participants who showed excessive head movement (see section “MR preprocessing and first-level model”), low familiarity with task stimuli, or timing or content mismatch issues in performance (see section “Stimulus familiarity and performance assessment”) were excluded as outliers. Demographic and performance details on accepted participants are summarized in Table 2; for age and gender distributions, see Figure 2. Performance was not associated with age apart from timing in Improvisation of new proverbs, where older age was weakly associated with a higher number of errors, *r_s_* (90) = 0.269, *p* = 0.010.

**Table 2:** Samples accepted for group analyses by task.

Task	*N* ^a^	Age^b^	Gender^c^	Education years^b^^,^^d^	Musical hobbies (%)^b^^,^^e^	Stimulus familiarity^b^^,^^f^	Timing errors^b^^,^^g^	Content errors^b^^,^^g^
**Repetition**								
Proverb and song	89 (100)	50.8 (16.9)	48/41/0	16.6 (4.5)	33.9 (29.6)	3.8 (0.2)	0.0 (0.1)	0.0 (0.1)
Proverb	100 (100)	49.2 (17.5)	55/45/0	16.5 (4.3)	33.1 (29.6)	3.9 (0.2)	0.0 (0.2)	0.0 (0.1)
Song	89 (100)	50.8 (16.9)	48/41/0	16.6 (4.5)	33.9 (29.6)	3.7 (0.4)	0.0 (0.0)	0.0 (0.1)
**Completion**								
Proverb and song	85 (100)	50.3 (16.9)	48/37/0	16.7 (4.6)	33.1 (29.8)	3.7 (0.2)	0.0 (0.2)	0.2 (0.4)
Proverb	99 (100)	49.1 (17.6)	55/44/0	16.5 (4.3)	33.0 (29.7)	3.9 (0.2)	0.0 (0.2)	0.1 (0.4)
Song	85 (100)	50.3 (16.9)	48/37/0	16.7 (4.6)	33.1 (29.8)	3.6 (0.3)	0.0 (0.2)	0.3 (0.7)
**Improvisation**								
Proverb and song	87 (100)	48.6 (17.6)	48/39/0	16.5 (4.4)	34.0 (30.0)	–	0.3 (0.5)	–
Proverb	92 (100)	48.4 (17.4)	50/42/0	16.5 (4.3)	33.4 (30.0)	–	0.5 (1.0)	–
Song	92 (100)	49.4 (17.6)	51/41/0	16.4 (4.4)	33.3 (30.0)	–	0.1 (0.2)	–

Demographic information of accepted participants with stimulus familiarity assessment results and performance outcomes by task. Conjunction proverb and song includes participants accepted in both vocal modalities within a task. Samples were based on maximal numbers of acceptable data.

a Sample size in group analyses after the exclusion of outliers reported as selected (original).

b Values reported as mean (SD).

c Female/male/other.

d Total years from the beginning of elementary school or equivalent.

e Percentage of lifetime experience with musical hobbies: years active with musical hobbies were divided by age to correct age-dependence in hobby years across the age range.

f Likert scale 1–4 (1 = completely unfamiliar, 4 = extremely familiar).

g Performance in fMRI tasks: erroneous trials per 20 trials in a task.

### Main effects in speech and singing production

**Figure 3:**
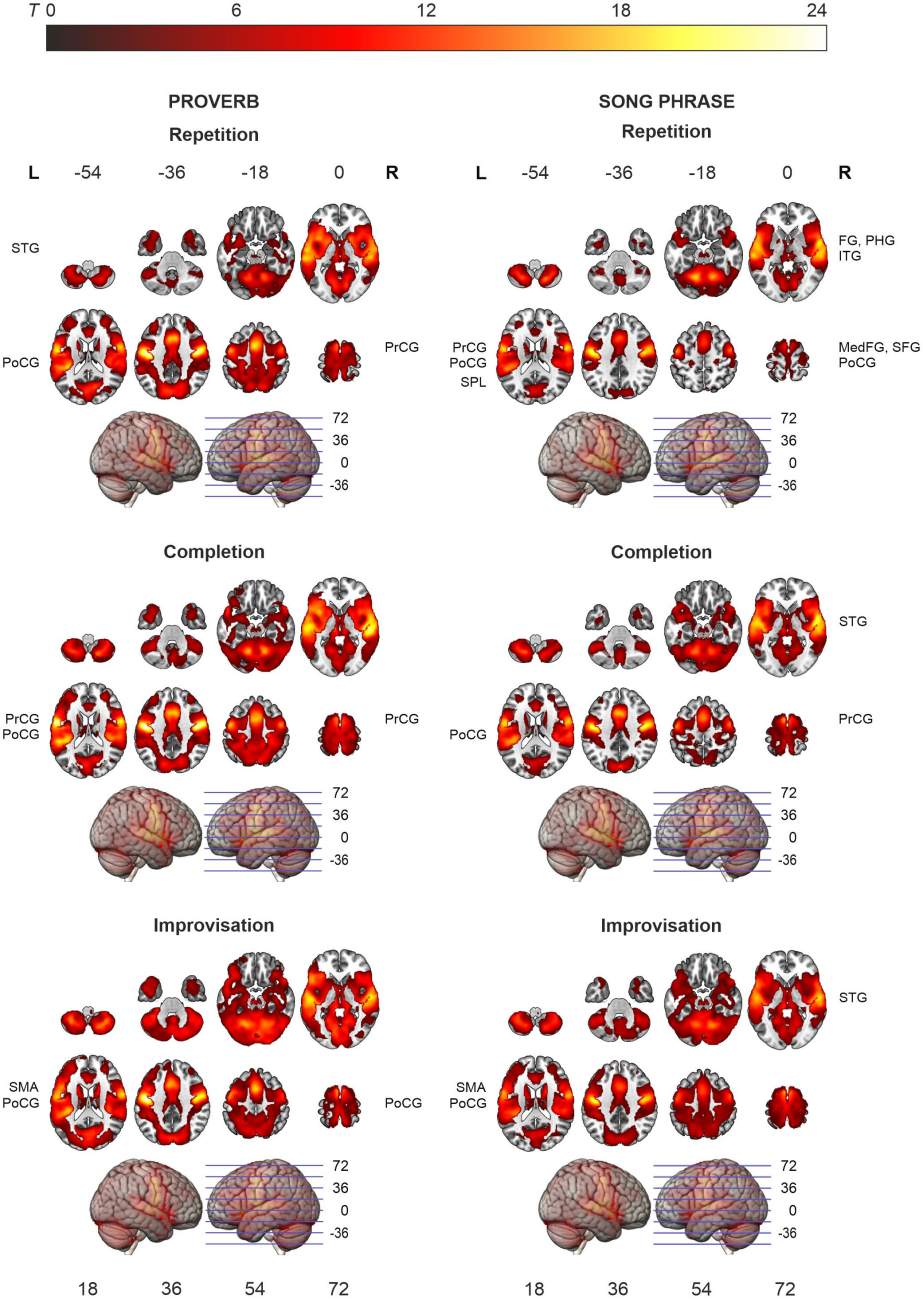
Activation maps of the main effects of speech (proverb) and singing (song phrase) production. Significant activity during production, thresholded over whole brain at *p* < 0.001 (uncorrected). Statistical significance corrected for familywise error (FWE) at cluster-level. Highest *T* values displayed in yellow to white tones.

**Figure 4:**
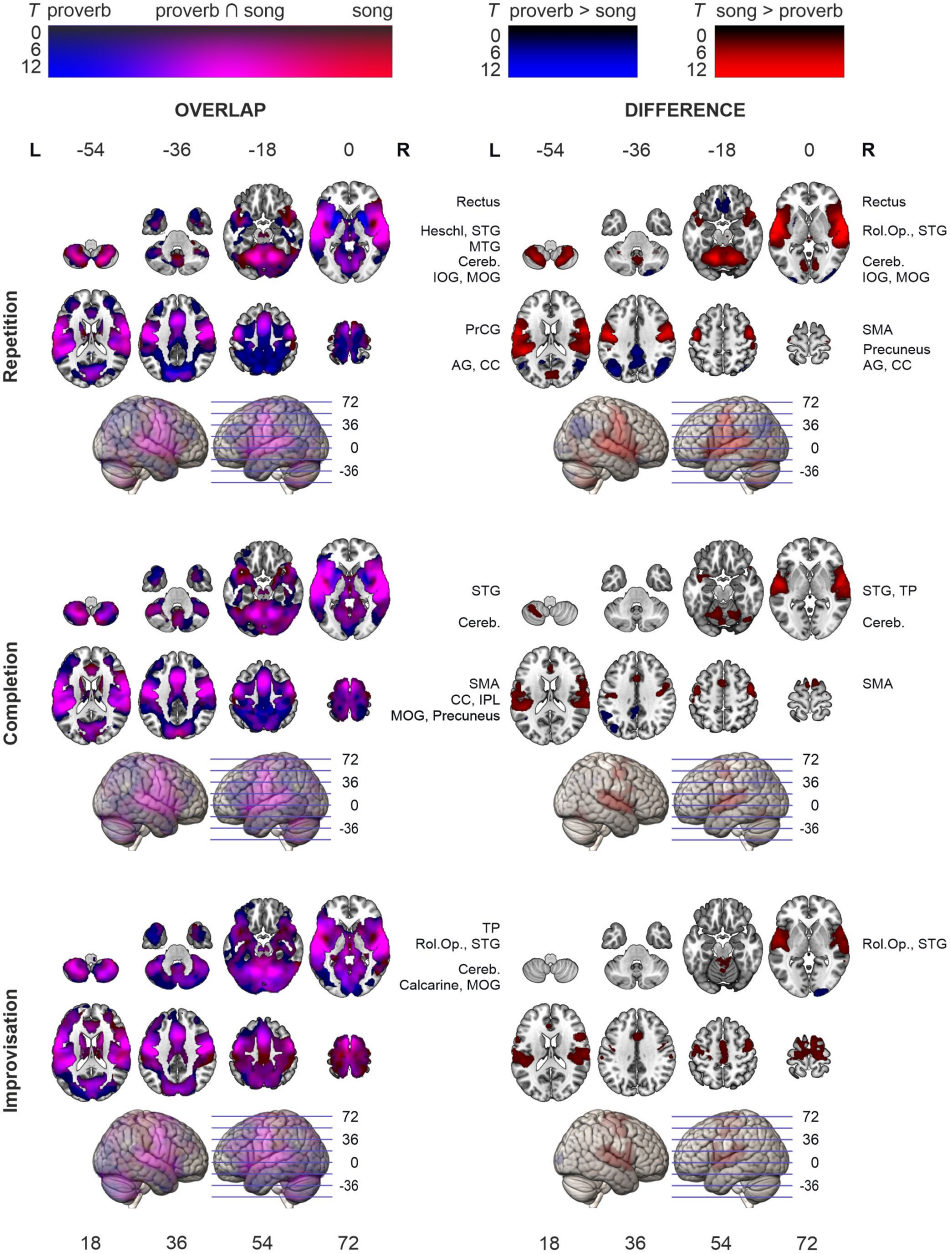
Activation maps comparing speech (proverb) and singing (song phrase) production. Overlap of speech and singing displayed in the left panel and their differences (speech > singing; singing > speech) on right. Significant activity during production, thresholded over whole brain at *p* < 0.001 uncorrected). Statistical significance corrected for familywise error (FWE) at cluster-level. Highest *T* values displayed in bright colours.

The *t*-tests showed that the neural processing of overt speech (Proverb tasks) and singing (Song tasks) elicited large activation clusters extending over frontal, temporal, parietal, and cerebellar regions (Table 3; Figure 3). Speech and singing showed great overlap but also several focal differences across the brain (Tables 4, 5; Figure 4). Specifically, compared to singing, speech showed stronger activation for Repetition in (i) the bilateral medial frontal gyrus (rectus), angular gyrus (AG), and middle occipital gyrus (MOG) as well as in the right precuneus and cerebellum (crus II); (ii) for Completion, increased activation was found in the left AG, MOG, and precuneus; and (iii) for Improvisation, in the bilateral MOG. Compared to speech, singing showed higher activation in the bilateral PrCG and PoCG, Rolandic operculum, STG, MTG, insula, supplementary motor area (SMA), IFG, MFG, supramarginal gyri, and cerebellum (with vermis) across all tasks.

**Table 3:** Main effects of speech (proverb) and singing (song phrase) production.

Task	Hemisphere	*p* (FWE)	Cluster size (voxels)	*T*	Peak coordinates		
					*x*	*y*	*z*
**Proverb**							
**Repetition**							
PoCG	L	<0.001	104,762	24.129	−46	−10	35
PrCG	R			23.776	46	−8	37
STG	L			20.626	−56	−16	5
**Completion**							
PoCG	L	<0.001	116,671	27.441	−50	−8	33
PrCG	L			25.223	−56	−2	21
PrCG	R			24.947	46	−10	37
**Improvisation**							
PoCG	L	<0.001	130,584	25.883	−46	−10	35
SMA	L			20.619	−2	8	61
PoCG	R			20.240	50	−6	31
**Song**							
**Repetition**							
PoCG	L	<0.001	68,993	21.754	−50	−8	33
PrCG	L			19.106	−52	−6	47
PoCG	R			18.763	54	−4	27
PrCG	L	<0.001	725	6.660	−20	−28	57
SPL	L			4.741	−32	−46	67
SPL	L			4.405	−22	−60	67
ITG	R	0.010	399	5.168	42	−2	−39
FG	R			4.735	26	4	−43
PHG	R			4.532	24	4	−27
MedFG	R	0.027	311	4.466	8	54	29
SFG	R			4.261	22	48	23
SFG	R			3.892	30	46	23
**Completion**							
PoCG	L	<0.001	92,172	21.835	−48	−10	35
PrCG	R			21.264	42	−10	37
STG	R			21.130	56	0	−3
**Improvisation**							
PoCG	L	<0.001	116,526	20.772	−48	−10	39
SMA	L			18.585	−2	4	69
STG	R			18.558	60	−22	5

FG, fusiform gyrus; ITG, inferior temporal gyrus; MedFG, medial frontal gyrus; PHG, parahippocampal gyrus; PoCG, postcentral gyrus; PrCG, precentral gyrus; SFG, superior frontal gyrus; SMA, supplementary motor area; SPL, superior parietal lobule; STG, superior temporal gyrus. Activation peaks within main effect clusters, thresholded over whole brain at *p* < 0.001 (uncorrected). Peak coordinates (mm) in MNI space. Statistical significance corrected for familywise error (FWE) at cluster-level.

**Table 4:** Main differences between speech (proverb) and singing (song phrase) production.

Task	Hemisphere	*p* (FWE)	Cluster size (voxels)	*T*	Peak coordinates		
					*x*	*y*	*z*
**Proverb > song**							
**Repetition**							
AG	L	<0.001	1954	7.639	−38	−72	37
AG	L			5.488	−40	−54	29
MTG	L			5.133	−52	−64	21
AG	R	<0.001	2,275	7.222	40	−72	37
AG	R			6.631	46	−68	31
MOG	R			5.830	50	−66	23
CC	R	<0.001	2,344	6.827	2	−38	35
Precuneus	R			5.761	2	−54	41
CC	L			5.707	−2	−32	41
Rectus	R	<0.001	876	4.899	6	24	−19
Rectus	L			4.755	−10	38	−19
Rectus	R			4.728	6	32	−21
Cerebellum (crus 2)	R	0.016	326	4.861	28	−88	−39
Cerebellum (crus 2)	R			4.610	44	−78	−41
Cerebellum (crus 2)	R			3.883	14	−92	−37
IOG	R	0.004	447	4.430	46	−86	−5
IOG	R			4.303	40	−92	3
MOG	R			4.219	32	−98	9
IOG	L	0.026	290	4.183	−46	−86	−7
MOG	L			3.837	−32	−98	−3
IOG	L			3.550	−22	−96	−7
**Completion**							
MOG	L	<0.001	1,371	5.043	−42	−76	33
IPL	L			4.985	−38	−76	41
IPL	L			4.885	−52	−58	43
CC	L	0.014	335	4.768	−2	−42	39
Precuneus	L			3.582	−6	−52	35
CC	L			3.483	−8	−52	27
**Improvisation**							
MOG	R	0.002	509	5.939	28	−98	5
Calcarine	R			5.487	14	−102	1

Activation peaks within difference clusters in speech > singing, thresholded over whole brain at *p* < 0.001 (uncorrected). Peak coordinates (mm) in MNI space. Statistical significance corrected for familywise error (FWE) at cluster-level.

**Table 5:** Main differences between singing (song phrase) and speech (proverb) production.

Task	Hemisphere	*p* (FWE)	Cluster size (voxels)	*T*	Peak coordinates		
					*x*	*y*	*z*
**Song > proverb**							
**Repetition**							
STG	R	<0.001	12,967	15.482	54	0	−3
STG	R			14.311	62	−16	1
Rolandic operculum	R			13.432	62	−8	7
STG	L	<0.001	12,053	15.140	−64	−24	9
Heschl	L			14.712	−60	−12	7
PrCG	L			14.004	−52	−6	47
Cerebellum (6)	L	<0.001	11,060	13.211	−16	−60	−21
Cerebellum (6)	R			11.738	18	−62	−21
Cerebellum (8)	L			9.447	−22	−62	−55
SMA	R	<0.001	3,271	8.154	2	10	63
CC	L/R			7.529	0	20	33
SMA	R			5.837	8	10	71
**Completion**							
STG	R	<0.001	6,540	11.770	52	−8	−1
TP	R			10.390	58	6	−3
STG	R			9.332	62	−18	5
STG	L	<0.001	5,470	10.655	−52	−8	1
STG	L			9.517	−62	−14	7
STG	L			7.717	−46	−38	15
SMA	R	<0.001	1,505	6.512	2	6	61
CC	L			4.906	−2	12	35
SMA	L			4.834	−4	4	73
Cerebellum (6)	L	<0.001	2,254	5.626	−12	−60	−19
Cerebellum (6)	R			5.129	16	−64	−15
Cerebellum (6)	L			4.959	−24	−62	−21
Cerebellum (8)	L	0.016	324	4.803	−22	−62	−59
Cerebellum (8)	L			4.694	−34	−52	−53
Cerebellum (8)	L	<0.001	6,540	4.616	−26	−58	−53
**Improvisation**							
STG	L	<0.001	11,408	8.252	−48	−10	−1
Rolandic operculum	L			8.103	−54	−4	1
STG	L			7.996	−60	−10	5
Rolandic operculum	R	<0.001	4,995	7.309	60	10	5
TP	R			7.174	60	8	−5
STG	R			7.104	62	−24	5
Cerebellum (3)	L	0.001	598	4.598	−4	−40	−15
Cerebellum (4–5)	L			4.193	−4	−60	−13
Cerebellum (3)	R			4.035	14	−38	−23

AG, angular gyrus; CC, cingulate cortex; IOG, inferior occipital gyrus; IPL, inferior parietal lobule; MOG, middle occipital gyrus; PrCG, precentral gyrus; SMA, supplementary motor area; STG, superior temporal gyrus; TP, temporal pole. Activation peaks within difference clusters in singing > speech, thresholded over whole brain at *p* < 0.001 (uncorrected). Peak coordinates (mm) in MNI space. Statistical significance corrected for familywise error (FWE) at cluster-level.

### Ageing effects in speech and singing

**Figure 5:**
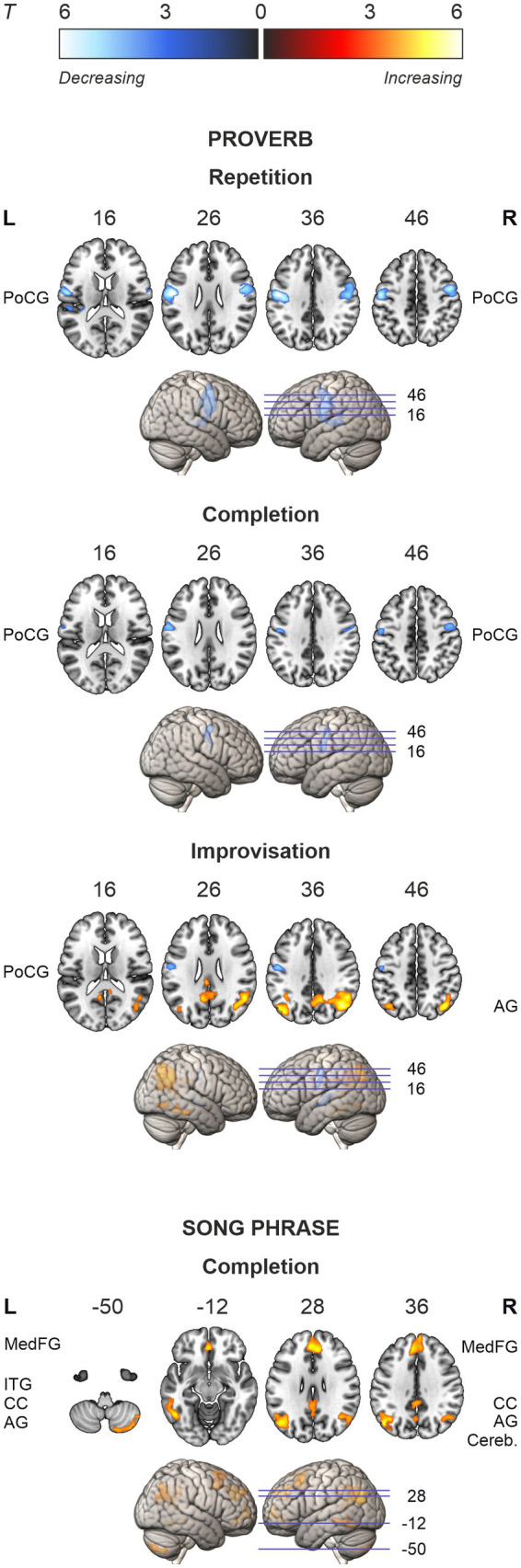
Activation maps of the ageing effects in speech (proverb) and singing (song phrase) tasks. Local activations decreasing and increasing as a function of age displayed in cold and hot colour bars, respectively. Highest *T* values displayed in light blue to white for decreasing activity and yellow to white for increasing activity. Thresholding was done over whole brain at *p* < 0.001 (uncorrected). Statistical significance was corrected for familywise error (FWE) at cluster-level.

The ageing effects in speech tasks are shown in Table 6 and Figure 5. The regression models showed that, in all speech (Proverb) tasks, activation in the left PrCG and PoCG decreased as a function of age; a similar effect was seen also in the right PrCG and PoCG for the Repetition and Completion tasks. In Proverb Repetition, activation decreased with ageing also in the left STG and the right MFG. In Proverb Improvisation, additional activation decrease with ageing was seen in the left STG. This was paralleled by increased activation with ageing in the right ITG and fusiform gyrus (FG), and also a trend towards increased activation in right IFG, which, however, did not survive familywise error correction (FWE *p* = 0.065).

**Table 6:** Ageing effects in speech (proverb) tasks.

Task	Hemisphere	*p* (FWE)	Association with age	Cluster size (voxels)	*T*	Peak coordinates		
						*x*	*y*	*z*
**PROVERB**								
**Repetition**								
PoCG	L	<0.001	Decrease	2071	7.136	−56	−8	23
PoCG	L				6.285	−52	−10	35
PoCG	L				5.847	−44	−14	33
MFG	R	<0.001	Decrease	1,505	6.892	46	−2	51
PoCG	R				4.880	46	−12	33
PoCG	R				4.873	50	−4	25
**Completion**								
PrCG	R	0.020	Decrease	311	4.968	46	−4	49
PoCG	R				3.697	58	−6	37
PoCG	L	0.009	Decrease	372	4.532	−60	−4	23
PoCG	L				4.250	−48	−10	37
PoCG	L				3.957	−54	−14	49
**Improvisation**								
PoCG	L	0.010	Decrease	374	5.124	−52	−10	41
PoCG	L				4.168	−54	−10	29
STG	L	0.038	Decrease	266	4.626	−46	−20	5
MTG	L				4.172	−52	−16	−5
STG	L				3.928	−50	−6	−7
AG	R	<0.001	Increase	2,701	5.723	42	−66	35
AG	R				5.202	52	−56	31
Precuneus	R				4.727	12	−56	33
MOG	L	0.001	Increase	583	4.794	−42	−74	35
AG	L				4.656	−50	−70	37
AG	L				4.025	−40	−66	47
FG	R	0.028	Increase	291	4.692	38	−60	−9
FG	R				4.473	28	−66	−9
ITG	R				3.822	52	−58	−5
ITG	R	0.035	Increase	272	4.524	52	−44	−15
ITG	R				4.215	56	−32	−19
ITG	R				3.947	58	−44	−9

AG, angular gyrus; FG, fusiform gyrus; ITG, inferior temporal gyrus; MFG, middle frontal gyrus; MOG, middle occipital gyrus; MTG, middle temporal gyrus; PoCG, postcentral gyrus; PrCG, precentral gyrus; STG, superior temporal gyrus. Activation peaks within ageing effect clusters in speech, thresholded over whole brain at *p* < 0.001 (uncorrected). Peak coordinates (mm) in MNI space. Statistical significance corrected for familywise error (FWE) at cluster-level. Association marked as increasing or decreasing with relation to advancing age.

In the singing (Song) tasks, no systematic ageing effects were found. Whole-brain analysis showed increasing activation with ageing in medial frontal, cingulate and bilateral parietal regions as well as bilateral prefrontal, left temporal and right cerebellar regions in the Song Completion task (Table 7; Figure 5). There were no significant ageing effects in the Song Repetition and Improvisation tasks.

**Table 7:** Ageing effects in singing (song phrase) tasks.

Task	Hemisphere	*p* (FWE)	Association	Cluster size (voxels)	*T*	Peak coordinates		
						*x*	*y*	*z*
**Song**								
**Completion**								
AG	L	<0.001	Increase	907	5.265	−48	−70	35
MTG	L				5.056	−50	−72	21
AG	L				4.951	−40	−66	33
ITG	L	0.011	Increase	364	4.944	−50	−58	−13
MTG	L				4.338	−60	−44	−9
MTG	L				4.231	−54	−36	−9
Cerebellum (crus 2)	R	0.020	Increase	318	4.894	38	−80	−47
Cerebellum (7b)	R				4.320	42	−66	−53
Cerebellum (8)	R				4.092	14	−78	−53
ACC	R	<0.001	Increase	927	4.742	4	40	27
MedFG	L				4.618	−6	38	37
MedFG	R				4.129	4	54	35
MedOG	L	0.010	Increase	412	4.554	−2	42	−9
MedOG	L				4.478	−2	60	−1
MedFG	L				3.516	−2	56	17
CC	L/R	0.002	Increase	543	4.553	0	−52	25
CC	L				4.443	−6	−44	11
CC	L/R				4.171	0	−40	39
MFG	L	0.023	Increase	309	4.395	−32	16	53
MFG	L				4.022	−42	20	45
AG	R	0.005	Increase	442	4.263	46	−68	43
AG	R				4.011	42	−60	25
AG	R				3.890	52	−62	29
SFG	R	0.003	Increase	490	4.160	30	12	65
MFG	R				4.116	36	14	43
SFG	R				3.828	22	24	57

ACC, anterior cingulate cortex; AG, angular gyrus; CC, cingulate cortex; ITG, inferior temporal gyrus; MedFG, medial frontal gyrus; MFG, middle frontal gyrus; MOG, middle occipital gyrus; MedOG, medial orbital gyrus; SFG, superior frontal gyrus. Activation peaks within ageing effect clusters in singing, thresholded over whole brain at *p* < 0.001 (uncorrected). Peak coordinates (mm) in MNI space. Statistical significance corrected for familywise error (FWE) at cluster-level. Association marked as increasing or decreasing with relation to advancing age.

### Difference of ageing effects in speech and singing

**Figure 6:**
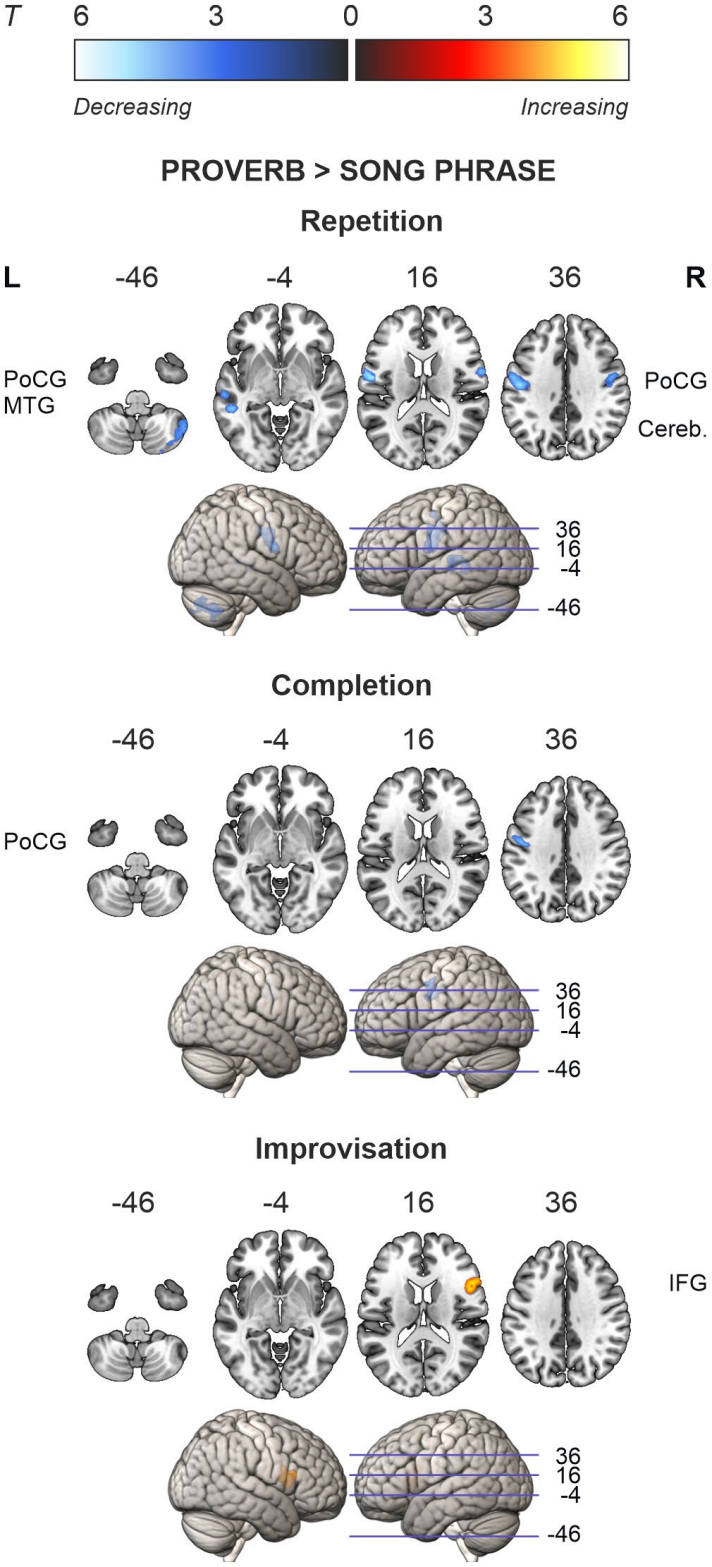
Ageing effects in the differences between speech (proverb) and singing (song phrase) tasks. Differences in local activations decreasing and increasing as a function of age displayed in cold and hot colour bars, respectively. Highest *T* values displayed in light blue to white for decreasing activity and yellow to white for increasing activity. Thresholding was done over whole brain at *p* < 0.001 (uncorrected). Statistical significance was corrected for familywise error (FWE) at cluster-level.

Regression models on the modality difference showed that the relative activation between speech and singing (Proverb > Song) decreased as a function of age in the left PrCG and PoCG in the Repetition and Completion tasks. In the Repetition task, there was also relative activation decrease with ageing in the right PrCG and PoCG as well as in the left STG and MTG. In the Improvisation tasks, the relative activation increased with ageing in the right IFG. By contrast, we found no ageing effects attributable to changes in singing (Song > Proverb). Results are outlined in Table 8 and Figure 6.

**Table 8:** Ageing effects in the differences between speech (proverb) and singing (song phrase) tasks.

Task	Hemisphere	*p* (FWE)	Association	Cluster size (voxels)	*T*	Peak coordinates		
						*x*	*y*	*z*
**Proverb > song**								
**Repetition**								
PoCG	L	<0.001	Decrease	711	6.377	−48	−10	27
PrCG	L				3.553	−48	−4	51
PrCG	R	0.004	Decrease	440	5.079	46	−6	29
PoCG	R				4.518	60	0	19
MTG	L	0.006	Decrease	414	4.712	−58	−28	5
MTG	L				4.302	−52	−40	−1
MTG	L				3.590	−50	−24	−7
Cerebellum (crus 2)	R	0.012	Decrease	348	4.064	50	−58	−47
Cerebellum (8)	R				3.871	42	−62	−57
Cerebellum (crus 2)	R				3.792	44	−70	−43
**Completion**								
PoCG	L	0.026	Decrease	265	4.637	−44	−8	33
PoCG	L				3.881	−56	−4	43
**Improvisation**								
IFG (oper.)	R	0.010	Increase	367	4.698	50	12	13
IFG (oper.)	R				3.624	48	10	21

IFG, inferior frontal gyrus; MTG, middle temporal gyrus; PoCG, postcentral gyrus; PrCG, precentral gyrus. Activation peaks within ageing effect clusters in the difference between singing and speech, thresholded over whole brain at *p* < 0.001 (uncorrected). Peak coordinates (mm) in MNI space. Statistical significance corrected for familywise error (FWE) at cluster-level. Association marked as increasing or decreasing with relation to advancing age.

### Ageing effects in lateralisation of speech and singing

**Figure 7:**
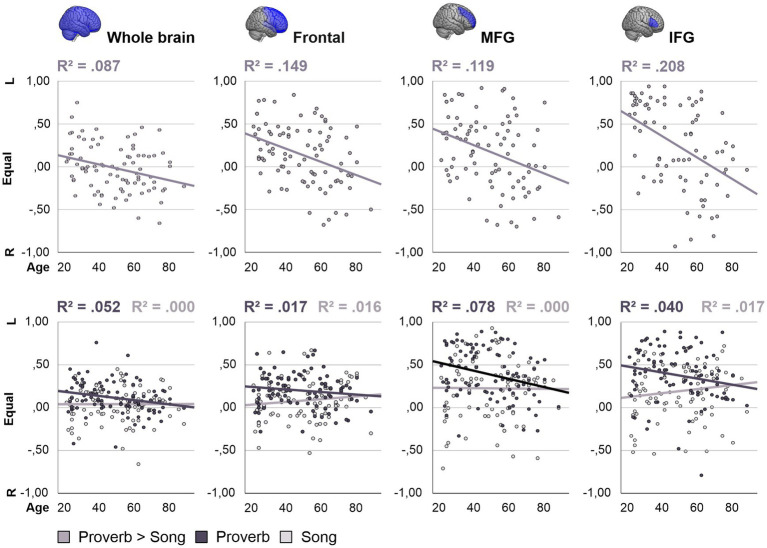
Ageing effects on laterality in the Improvisation tasks. Age-related changes in the laterality index (LI) displayed for whole-brain grey matter, frontal lobes, and middle (MFG) and inferior frontal gyri (IFG) bilaterally. Positive values suggest stronger contribution of the left-hemisphere region. For each region, age-related changes in the difference of speech and singing (Proverb > Song phrase) are displayed on top in middle grey. Respective age trends in improvised speech (Proverb) and singing (Song phrase) are shown below in dark and light grey, respectively.

Regression analyses on the LI showed that age predicted lateralisation change in Improvisation but not in other tasks. Here, age was the only significant predictor of LI in the stepwise regression. The results from Proverb and Song Improvisation are summarised in Figure 7. Over the whole brain, age predicted the laterality difference (indicated by LI) between speech and singing in the Improvisation task (Proverb > Song), *F* (1, 85) = 8.137, *p* = 0.005, *R*^2^ = 0.087. This effect was driven by speech, which showed increasing rightward lateralisation trend with older age, *F* (1, 90) = 4.968, *p* = 0.028, *R*^2^ = 0.052. After correction for multiple comparisons, the relative effect remained significant in the frontal lobes, *F* (1, 85) = 14.874, *p* < 0.001, *R*^2^ = 0.149, albeit with no significant source in either vocal modality at this level. *Post hoc* testing on the relative ageing effect (Proverb > Song) in pre-selected frontal sub-ROIs (IFG, MFG, OFG, PrCG) located sources in the MFG at *F* (1, 85) = 11.447, *p* < 0.001, *R*^2^ = 0.119, and IFG at *F* (1, 77) = 20.267, *p* < 0.001, *R*^2^ = 0.208; this trend was also significant in speech in the MFG, *F* (1, 90) = 7.578, *p* = 0.007, *R*^2^ = 0.078 (alpha levels corrected for multiple comparisons). No significant results were observed in singing.

## Discussion

This study investigated the intricacies of the ageing brain by mapping the neural differences between speech and singing across a diverse age range in healthy adults, under tasks with varying cognitive demands: repetition of familiar phrases, completion of familiar phrases, and improvisatory completion of new phrases.

Notably, our results revealed a systematic age-related decrease of activity in the left/bilateral precentral gyrus (PrCG) and postcentral gyrus (PoCG) during speech. Conversely, when participants were asked to improvise new proverbs, there was an increase in activation in the right inferior temporal gyrus (ITG) and fusiform gyrus (FG), bilateral angular gyrus (AG), and right prefrontal regions. As expected, the laterality of prefrontal activation shifted from left to right with advancing age. Singing, on the other hand, did not demonstrate a general ageing effect. However, when the task involved completing song phrases from memory, we observed increased activation in a widespread network, including the default mode network (DMN) and lateral prefrontal regions.

These findings shed new light on the complex interplay of speech and singing in the ageing brain and underscore the remarkable adaptability of our neural mechanisms across the lifespan.

### Main differences between the modalities

Consistent with previous findings, speech and singing tasks elicited highly overlapping activity, but with several focal differences. Compared to speech, singing tasks elicited stronger activation in prefrontal (IFG, MFG), auditory (STG, Heschl), somatosensory and motor (PrCG, PoCG, SMA), as well as associative regions (SMG, insula and Rolandic operculum) and the cerebellum, bilaterally. These regions fall under the complex bihemispheric fronto-temporo-parieto-cerebellar networks for cognitive and auditory-motor feedback control and somatosensory integration in vocal production (Callan et al., 2006; Özdemir et al., 2006; Zarate, 2013; Behroozmand et al., 2015), hence replicating a typical finding of their stronger bilateral engagement in singing (Callan et al., 2006; Özdemir et al., 2006; see also Jeffries et al., 2003).

Potentially owing to greater cognitive demands and sample size, the present results also showed focally stronger activity in speech compared to singing not reported in previous studies. It should also be noted that, in Finnish language, meaning is characteristically conveyed *via* phonological length while intonation carries little semantic value; this may introduce an exceptionally great gap between the auditory-motor processing of speech and singing. Compared to singing, repetition and completion of familiar proverbs elicited higher activity in the IPL and SPL regions (AG, precuneus), previously associated with attentional control in information retrieval (Emch et al., 2019), as well as with episodic memory and semantic cognition (Humphreys et al., 2021). Furthermore, in the Repetition task, we observed accompanying activation in the right crus II of the cerebellum, a region believed to play a role in linguistic processing, particularly in the prediction and error detection of phonological content (Lesage et al., 2017; see also Callan et al., 2007).

### Ageing effects in speech and singing

#### Reductions in somatomotor activation in speech

Compared to singing, activity in left/bilateral PoCG and PrCG decreased with advancing age in Repetition and Completion of familiar proverbs. Consistent across all Proverb tasks, this difference was likely driven by speech. Centred in the somatosensory regions, such activation reductions might reflect reduced ability to process somatosensory feedback to support vocal motor production. It should also be noted that no lateralisation changes were found in the somatomotor regions, which suggests that these decreases are not characterised by hemispheric asymmetry reduction.

In favour of this account, PoCG and adjacent regions belong to typical sites of accelerated grey matter (GM) loss, often displaying left bias (Di et al., 2014; Minkova et al., 2017; see also Solé-Padullés et al., 2009).Consistent with the present results, a consequence from this interpretation is that speech, relying more heavily on left hemisphere regions potentially showing faster age-related atrophy, would be subject to more abrupt adaptation than singing. This is not to say that ageing impacted the modalities selectively but, rather, that the differential distribution of singing networks would provide neural resources for more flexible (internal) adaptability. Indeed, a recent study on post-stroke aphasia (Pitkäniemi et al., 2023) reported that singing shares language network resources with speech but relies less on the dorsal pathway typically associated with sensory-motor mapping (e.g., Saur et al., 2008). Flowing across the somatomotor regions, this distinction might explain a more rapid age-induced need for reorganisation in speech than singing. In their controlled study on primary progressive aphasia (PPA), Grube et al. (2016) also speculated that auditory deficits across different subtypes of PPA may relate to processing along the dorsal stream, which might in turn help explain previous observations of dissociated speech and singing ability in PPA (e.g., Polk and Kertesz, 1993; see also Baird and Thompson, 2019). Future studies should aim to map the network characteristics during singing which may overcome processing deficits impairing speech in clinical populations and, potentially, delay the need for network reorganisation in healthy ageing. Moreover, understanding both pathological and age-associated network changes in speech versus singing could directly benefit clinicians designing rehabilitative strategies for aphasias such as PPA.

As the singing network is inevitably subject to structural declines in healthy ageing, another mechanism to consider is that neural differentiation, i.e., dispersion from specialised to more general-purpose networks following GM atrophy (see Shafto and Tyler, 2014), might take a different trajectory in singing. Thus, a secondary suggestion for future work is to assess whether the somatomotor network’s use of external connections (e.g., Geerligs et al., 2015; see also Carp et al., 2011), for instance, varies by higher vocal motor control demands involved in the melodic-lexical co-processing in singing.

#### DMN and prefrontal activation in recollection of song phrases

The sole ageing effect in singing was observed in Completion of song phrases from memory as increased activation in the medial frontal and parietal default mode network (DMN) regions, as well as bilateral MFG/SFG, left ITG/MTG, and right cerebellum. While these regions have been associated with recognition of familiar musical excerpts (Platel et al., 2003) and internal tasks such as retrieval of episodic and semantic knowledge (Smallwood et al., 2021), their increased activation in Completion but not in Repetition of familiar song phrases, specifically at an older age, seems to suggest a task-related effect.

Increased coupling of lateral prefrontal and DMN regions is thought to support cognitive performance by means of shifting from fluid abilities to higher reliance on experience (Turner and Spreng, 2015; Spreng and Turner, 2019), while increases in lateral cerebellar activation may support motor preparation for producing verbal content (Riecker et al., 2000; Tremblay et al., 2017). Indeed, this task elicited the highest number of average errors regarding a mismatch between target and response (Table 2). As the pattern did not appear in the singing versus speech comparison, this is suggestive of increased cognitive effort in retrieving a song phrase ending rather than representing an ageing effect related to singing or retrieval as such. Indeed, phrases belonging to the larger context of a song might be less automated than proverbs, which are complete linguistic entities on their own.

#### Parietal and posterior medial activation in improvised speech

Ageing elicited increasing activation in right FG and posterior ITG and bilaterally in the AG with right-hemisphere bias in improvisation of new proverbs. These regions are typically associated with semantic tasks in the young adult brain (Benedek et al., 2014; Bonilha et al., 2017; Liu et al., 2021). Consistent with the task demands of Proverb Improvisation, age-related volume losses in FG and ITG have been associated with declines in verbal fluency, verbal memory, attention, and executive function (Armstrong et al., 2020; see also Spreng and Turner, 2019). Recent work (Adnan et al., 2019a,b; Martin et al., 2023) also suggests some involvement of FG and posterior ITG as a part of large-scale network reorganisation supporting semantic/creative cognition at older age, where bilateral FG may expand their communication network while other regions serve as additional connector hubs (Martin et al., 2023). Speculatively, the observed activation increases might thus represent successful functional compensation in response to cognitive task demands and ongoing network reorganisation.

#### Prefrontal activation in improvised speech

In the Improvisation tasks, difference between the vocal modalities (speech > singing) showed an age-associated increase in activation in the right IFG (opercularis, triangularis) with a respective reduction of left bias in the laterality (LI) of IFG activity. These effects were potentially driven by a marginally significant increase of right IFG activation in speech—a classic example of prefrontal hemispheric asymmetry reduction in older adults (Cabeza, 2002) previously observed during rapid naming, for instance (Hoyau et al., 2017). Separate analyses of laterality confirmed minor rightward shifts across the whole brain and in the prefrontal regions such as MFG with older participants showing relatively increased use of right-hemisphere homologues in speech. By contrast, age was not associated with laterality in singing. Providing support for our last hypothesis, these results suggest that, under similar task demands, speech and singing show dissociative ageing effects on prefrontal hemispheric asymmetry.

## Conclusion

The present study found two-fold main effects of age in speech: systematic decreases of left somatosensory activity, likely related to local network reorganisation, and rightward shifts of improvisation-induced activation in the prefrontal regions. Interestingly, the former pattern was not observed in singing, which may be due to differential network reorganisation at older age. The latter set of findings, on the other hand, corroborate previous reports of reduced hemispheric asymmetry with advancing age in speech (Cabeza, 2002; Reuter-Lorenz and Park, 2014). Consistent with the present improvisation task design, this mechanism is typically observed in the prefrontal regions during tasks engaging working memory and fluid ability (Höller-Wallscheid et al., 2017; Hennessee et al., 2022), for instance. The expected null effects from improvisation of new song phrases suggests that singing, which exhibits more balanced activation than speech between the hemispheres at a young age (Callan et al., 2006; Özdemir et al., 2006), may also require less cross-hemispheric adaptation later in life.

However, we would like to acknowledge some limitations of the present study that merit future research. First, the present study is cross-sectional, which hinders the possibility of tracing individual ageing trajectories and their contributions at population level. Second, the present study derived stimuli from naturalistic materials for higher ecological validity in the memory tasks; future studies investigating ageing mechanisms in singing should employ one-to-one matched speech and song stimuli to rule out the possibility of a stimulus effect.

In conclusion, we have shown systematic ageing effects in speech production that did not appear in singing. These novel results challenge the current functional models of ageing and highlight the importance to consider the value of null effects alongside well-established ageing effects. Further, these findings are an important step towards understanding the positive effects of singing on speech in healthy ageing, as well as its rehabilitative potential in neurodegenerative disorders such as PPA. In future, it would be interesting to explore how age-related functional changes in speech and singing processing are linked to structural neural changes, such as grey matter volume and white matter connectivity, and whether similar effects are seen also in age-related neurological disorders.

## Data availability statement

The raw data supporting the conclusions of this article will be made available by the authors, without undue reservation.

## Ethics statement

The studies involving humans were approved by the European Research Council Executive Agency (ERCEA) and the University of Helsinki Ethical Review Board in the Humanities and Social and Behavioural Sciences. The studies were conducted in accordance with the local legislation and institutional requirements. The participants provided their written informed consent to participate in this study.

## Author contributions

All authors contributed to the conception and design of the study. JK programmed the fMRI tasks. NM-M, AS, BK, and TS supervised the fMRI analyses. TS handled project administration and funding acquisition. NM collected the data, carried out the analyses, and wrote the first draft of the manuscript. All authors contributed to the writing, review, and editing of the manuscript and approved the submitted version.

## Funding

Financial support for the work was provided by the Finnish Cultural Foundation (grant no. 00210736), Academy of Finland (grant no. 346211), and the European Research Council (grant no. 803466).

## Conflict of interest

The authors declare that the research was conducted in the absence of any commercial or financial relationships that could be construed as a potential conflict of interest.

## Publisher’s note

All claims expressed in this article are solely those of the authors and do not necessarily represent those of their affiliated organizations, or those of the publisher, the editors and the reviewers. Any product that may be evaluated in this article, or claim that may be made by its manufacturer, is not guaranteed or endorsed by the publisher.
